# Enhanced Color Nighttime Light Remote Sensing Imagery Using Dual-Sampling Adjustment

**DOI:** 10.3390/s25072002

**Published:** 2025-03-22

**Authors:** Yaqi Huang, Yanling Lu, Li Zhang, Min Yin

**Affiliations:** 1 College of Geomatics and Geoinformation, Guilin University of Technology, Guilin 541004, China; 2120221988@glut.edu.cn (Y.H.); 6612012@glut.edu.cn (L.Z.); 2007019@glut.edu.cn (M.Y.); 2 Guangxi Key Laboratory of Ecological Spatio-Temporal Big Data Perception, Guilin 541004, China

**Keywords:** up-sampling, down-sampling, NPP/VIIRS, Landsat, image fusion, color nighttime light, urban functional division

## Abstract

Nighttime light remote sensing imagery is limited by its single band and low spatial resolution, hindering its ability to accurately capture ground information. To address this, a dual-sampling adjustment method is proposed to enhance nighttime light remote sensing imagery by fusing daytime optical images with nighttime light remote sensing imagery, generating high-quality color nighttime light remote sensing imagery. The results are as follows: (1) Compared to traditional nighttime light remote sensing imagery, the spatial resolution of the fusion images is improved from 500 m to 15 m while better retaining the ground features of daytime optical images and the distribution of nighttime light. (2) Quality evaluations confirm that color nighttime light remote sensing imagery enhanced by dual-sampling adjustment can effectively balance optical fidelity and spatial texture features. (3) In Beijing’s central business district, color nighttime light brightness exhibits the strongest correlation with business, especially in Dongcheng District, with r = 0.7221, providing a visual tool for assessing urban economic vitality at night. This study overcomes the limitations of fusing day–night remote sensing imagery, expanding the application field of color nighttime light remote sensing imagery and providing critical decision support for refined urban management.

## 1. Introduction

Nighttime light remote sensing plays a significant role in various fields due to its ability to reflect human activity characteristics through the intensity of nighttime light [1,2,3]. With the study of DMSP/OLS (Defense Meteorological Satellite Program/Operational Linescan System) and NPP/VIIRS (National Polar-orbiting Partnership/Visible Infrared Imaging Radiometer Suite), China’s launch of the Luojia-1 satellite has achieved a significant breakthrough in obtaining high-resolution nighttime light remote sensing data. The continuity and objective independence advantages of nighttime light remote sensing make it closely related to human activities on the Earth’s surface. Therefore, nighttime light remote sensing imagery can provide an important data source for earthquake disaster assessment, power consumption, light pollution, carbon emission assessment, and other fields [4,5,6,7]. However, traditional nighttime light remote sensing imagery has the limitations of single band and low resolution, due to which it cannot truly capture the ground surface. Therefore, color nighttime light remote sensing has been produced. It achieves a spatial resolution ranging from kilometers to meters thanks to spectral imaging technology, and spectral information has also developed from single-band black-and-white images to multi-band color images. This enables color nighttime light remote sensing to perform an important function in surface environmental monitoring.

In 2017, China’s independently developed Jilin-1 satellite (JL1-3B) was able to obtain high-resolution (1 m) color nighttime light remote sensing imagery which can better monitor and analyze urban nighttime light [8,9]. For example, Watson C. S. et al. conducted a correlation analysis between Jilin-1 data and economic development, finding that the brightness of urban nighttime lights determines the level of economic development [10]. Guk E et al. conducted a correlation analysis of urban nighttime light types using different spectral indices and found that compared to the red and green bands, the correlation between the blue band and light intensity is the lowest [11]. In order to better promote the achievement of sustainable development goals, in 2021, China launched the Sustainable Development Goals Satellite 1 (SDGSAT-1), a satellite specifically designed to collect data related to sustainable development [12,13]. Its onboard low-light imager can capture the distribution of color nighttime light on the Earth’s surface at night. Guo et al. used SDGSAT-1 to study the spatial differences between spectral bands and light intensity in land use, revealing that spectral bands can influence land use conditions [14]. Zhang et al. developed a denoising algorithm for SDGSAT-1, effectively removing striping noise and salt-and-pepper noise from images [15]. However, color nighttime light remote sensing imagery has high design and operation costs, and there are still issues such as limited data coverage, inconvenient access, and high cost, which seriously restrict its global application. Nowadays, nighttime light remote sensing can fully reflect the distribution of regional light, and daytime optical images can reflect ground information. So, a combination of the two can provide a more comprehensive analysis of urban functional types [16,17]. Thus, image fusion technology provides a practical solution. It is the process of combining data from different sensors to create a new fused image. Different fusion algorithms have their own advantages and weaknesses. Common fusion methods include super-resolution Bayesian pansharp, intensity hue saturation (IHS), and principal component change (PCA) [18,19,20,21,22]. However, the resolution of daytime and nighttime remote sensing imagery has a large gap, and it is necessary to sample images with the appropriate resolution to obtain the best fusion image. Therefore, it is of great significance to effectively sample daytime and nighttime remote sensing imagery.

Based on the above problems, a dual-sampling adjustment enhancement method is proposed based on the existing fusion algorithm. The method generates color nighttime light remote sensing imagery by fusing daytime optical images with nighttime light remote sensing imagery. Up–down dual sampling modes are used for comparative analysis, which verifies the effectiveness of the dual-sampling adjustment in enhancing color nighttime light remote sensing imagery. This provides an important reference basis for the study of urban functional types.

## 2. Research Area and Data Processing

### 2.1. Research Area

As the capital of the People’s Republic of China, Beijing is an important center for politics, culture, and the economy. It is also a key transportation hub and a popular tourist destination in the country. Located on the northern edge of the North China Plain, its geographical coordinates range from 115°20′ to 117°30′ East and from 39°28′ to 41°05′ North. The region is densely populated and is one of the representative cities of high-level urbanization development.

### 2.2. Data Processing

**Figure 1:**
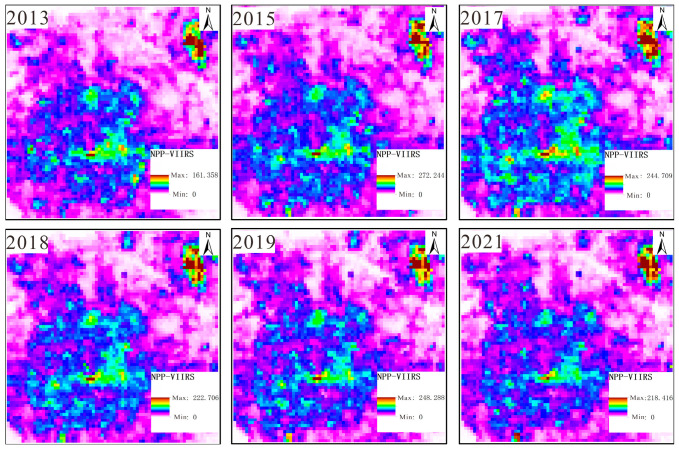
Nighttime light remote sensing imagery dataset.

**Figure 2:**
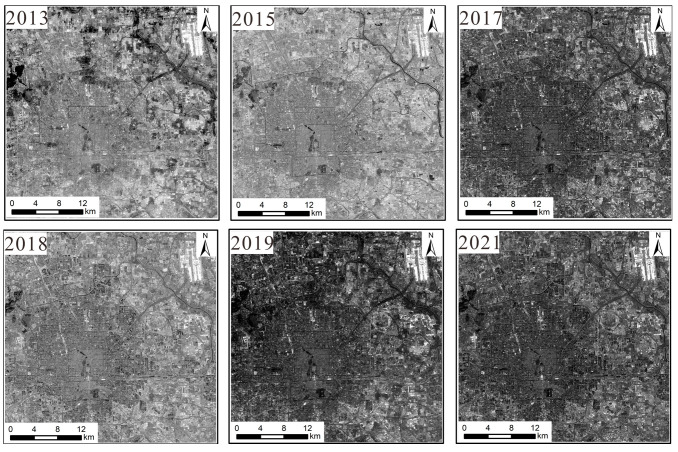
Panchromatic image dataset.

**Figure 3:**
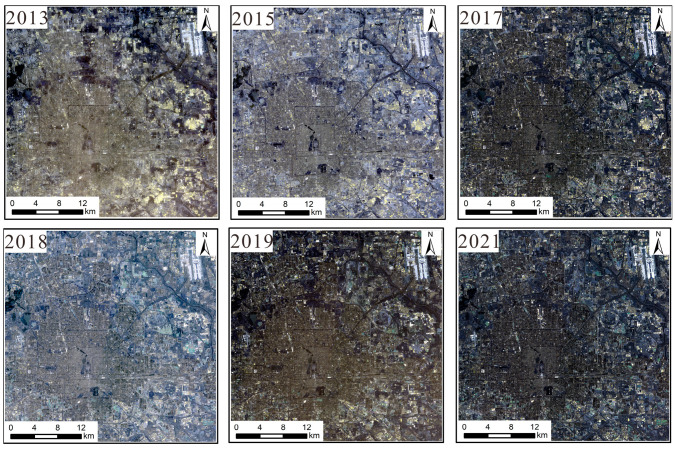
Multispectral image dataset.

(1) NPP/VIIRS data: These data come from the long-term nighttime light dataset for China published by the Global Change Science Research Data Publishing System [23] and from the National Geophysical Data Center (NGDC) under the National Oceanic and Atmospheric Administration (NOAA) of the United States. Compared to DMSP/OLS nighttime light remote sensing, the cloud-free monthly data from NPP-VIIRS with 500 m spatial resolution do not have the problem of pixel value saturation. Moreover, the monthly data have completely eliminated the influence of moonlight, aurora borealis, and other stray light sources, and they effectively monitor the ground nighttime light situation. The steps of data processing were as follows: The Chinese administrative division vector data extracted by the mask were used to obtain nighttime light remote sensing images of Beijing during 2013–2021. The data were projected onto the WGS1984 geographic coordinate system and the Lambert Equal Area projection coordinate system. The processing of data for the six-year period was completed as shown in Figure 1.
(2) Landsat 8 data: These data were sourced from the Geospatial Data Cloud. This refers to Landsat 8OL-TIRS (OLI = Operational Land Image; TIRS = Thermal Infrared Sensor) images covering Beijing for the period from 2013 to 2021, where OLI is mainly in the visible to short-wave infrared bands and TIRS is in the thermal infrared band. This study uses OLI data, which contain multispectral images with a resolution of 30 m and panchromatic images with a resolution of 15 m. The radiometric calibration removes the error caused by the difference in sensor response; the atmospheric correction further removes the atmospheric scattering and absorption effects to obtain the true reflectance of the ground surface, and the panchromatic images are radiometrically calibrated to correct the radiometric distortion caused by atmospheric effects to ensure the clarity of spatial details. The fidelity of the pre-processed images is greatly improved, and the color distortion is reduced during fusion. Finally, the images are mosaicked and extracted from the regional map to obtain the multispectral images and panchromatic remote sensing images for 2013–2021. The results are shown in Figure 2 and Figure 3.

## 3. Dual-Sampling Adjustment to Generate Color Nighttime Light Remote Sensing Imagery

### 3.1. Dual-Sampling Adjustment Method

(1) Down-sampling adjustment

Due to the differences in imaging time and sensors between daytime and nighttime remote sensing imagery, the 30 m multispectral image (Landsat_MS) is first down-sampled to 500 m to match the nighttime light remote sensing imagery. The red and green bands (R, G) are fused with NPP-VIIRS (B) to obtain the 500 m composite image 1 (R, G, B) by using the band stacking method. To maintain consistency with the original daytime optical image, the bicubic convolution method is used to up-sample it to a 30 m resolution to obtain composite image 2, ensuring it maintains an integer multiple relationship with the 15 m panchromatic image.

(2) GS transformation

Gram–Schmidt (GS) Pan Sharpening is a method that uses multidimensional linear orthogonal transformations to process images, reducing information redundancy while better preserving spectral characteristics and detailed texture information [24,25]. The essence lies in using GS orthogonalization to decompose the information of synthetic image 2 (R, G, B30m) into a linear combination of orthogonal basis vectors. By applying GS transformation to synthetic image 2 with a spatial resolution of 15 m, a 15 m fused image is generated.

(3) High-pass filter enhancement

**Figure 4:**
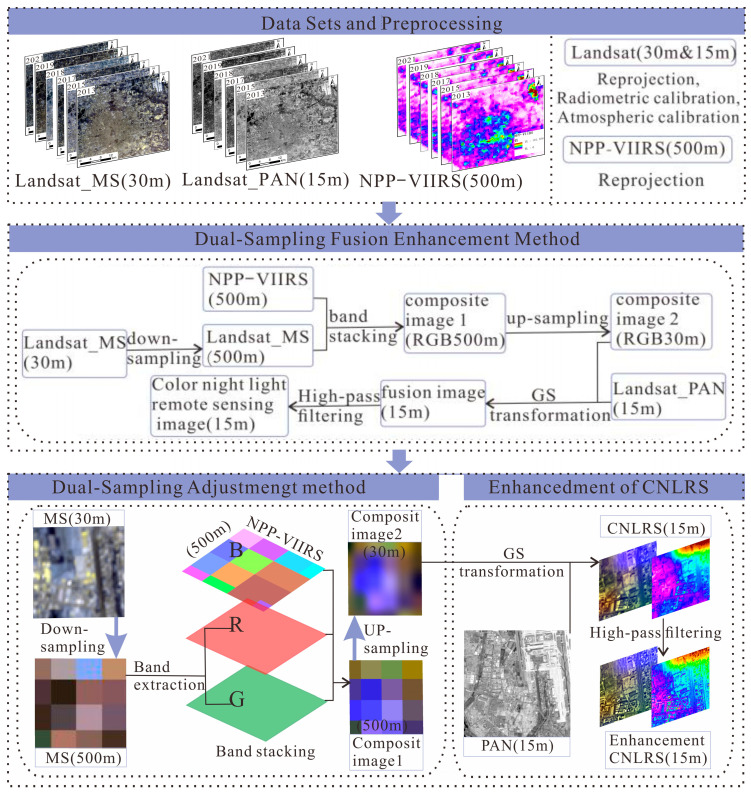
Implementation of the dual-sampling adjustment image fusion method.

High-pass filter enhancement allows high-frequency signals to pass through while weakening and preventing low-frequency signals, achieving an enhancement of specific frequencies in images. Its advantage lies in the ability to precisely control image information based on different frequencies and specific needs, thereby optimizing image quality and highlighting detailed features. The formula is as follows [26,27]:

(1) $$H\left(u,v\right)=\left\{\begin{array}{c} 0,D\left(u,v\right)\leq D_{0}\\ 1,D\left(u,v\right)>D_{0} \end{array}\right.$$

where H(u,v) means the frequency of the high-pass wave,$D\left(u,v\right)={\left(u^{2}+v^{2}\right)}^{1/2}$ denotes the distance from the point $\left(u,v\right)$ to the origin, and $D_{0}$ denotes the distance from the as-of-frequency point to the origin. When $D\left(u,v\right)$ is greater than 0, the filter coefficient is taken as 1, which represents the enhancement of high-frequency information; when it is less than 0, the filter coefficient is taken as 0, which means that the low-frequency information is weakened. The specific process is shown in Figure 4.

**Figure 5:**
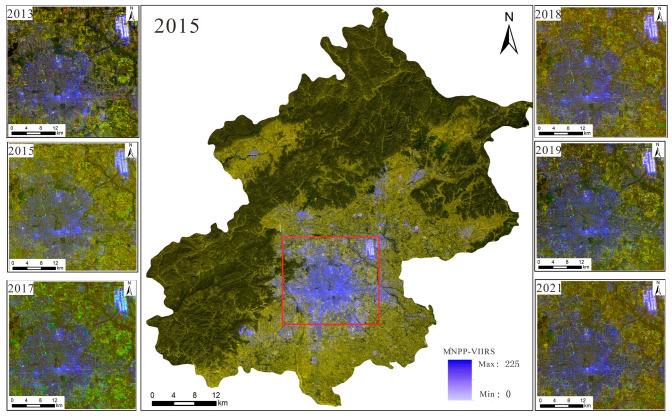
Dual-sampling adjustment method for fusion generation of color nighttime light remote sensing imagery.

**Figure 6:**
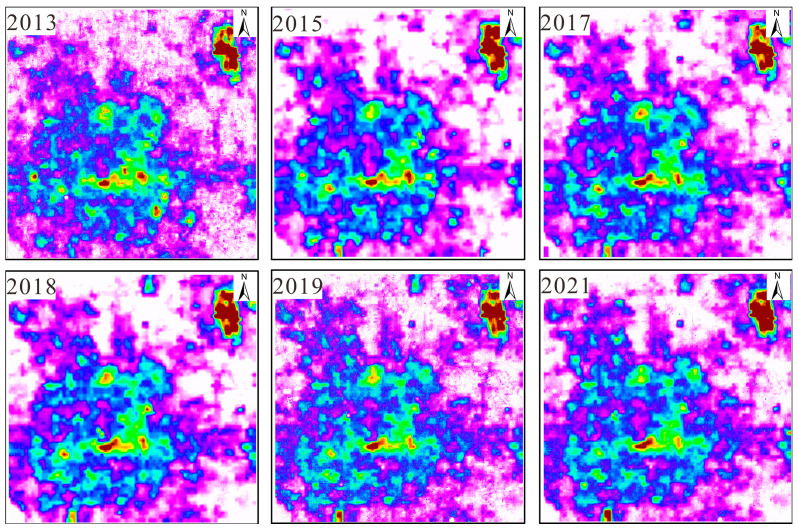
The third band of color nighttime light remote sensing imagery.

(4) Based on the above methods, color nighttime light remote sensing imagery of Beijing for 2013, 2015, 2017, 2017, 2019, and 2021 was generated. The results are shown in Figure 5 and Figure 6.

From 2013 to 2021, the distribution of nighttime light in the central area of Beijing remained relatively stable. Not only can one directly observe the distribution of nighttime lights in the city, represented by the purple-covered areas, but one can also see the information about the objects covered by the nighttime light. For example, the most central urban area usually shows a significant brightness of light, while the old city areas may exhibit different light distribution due to the renovation of lighting facilities.

As shown in Figure 6, the brightness of light in the center of Beijing tends to gradually weaken as the area expands. For example, commercial centers and transportation facilities exhibit brighter light, while residential areas and surrounding green spaces show weaker light. Moreover, the farther away from the city center, the gradually weaker the light brightness becomes.

### 3.2. Sampling Comparison Method

To evaluate the effectiveness of dual-sampling adjustment on image quality, this paper compares it with single up-sampling and down-sampling using NND transformation, Brovey transformation, PCA transformation, and GS transformation for fusion to obtain color nighttime light remote sensing imagery. The specific methods are discussed below.

(1) Down-sampling multispectral image

In order to keep the same resolution with the 500 m nighttime light remote sensing image, the 30 m multispectral image is down-sampled, and its red and green bands are selected to be fused with the nighttime light remote sensing image to obtain the 500 m color nighttime light remote sensing image. The color nighttime light remote sensing image is fused with the 15 m panchromatic image by using a specific fusion algorithm.

(2) Up-sampling adjustment

Bicubic convolution resampling is performed on the 500 m spatial resolution nighttime light remote sensing image to match the 30 m spatial resolution multispectral image. The red and green bands are extracted and fused with the nighttime light remote sensing image to obtain the 30 m color nighttime light remote sensing image, and then it is fused with a 15 m panchromatic image.

(3) Dual-sampling multispectral image

**Figure 7:**
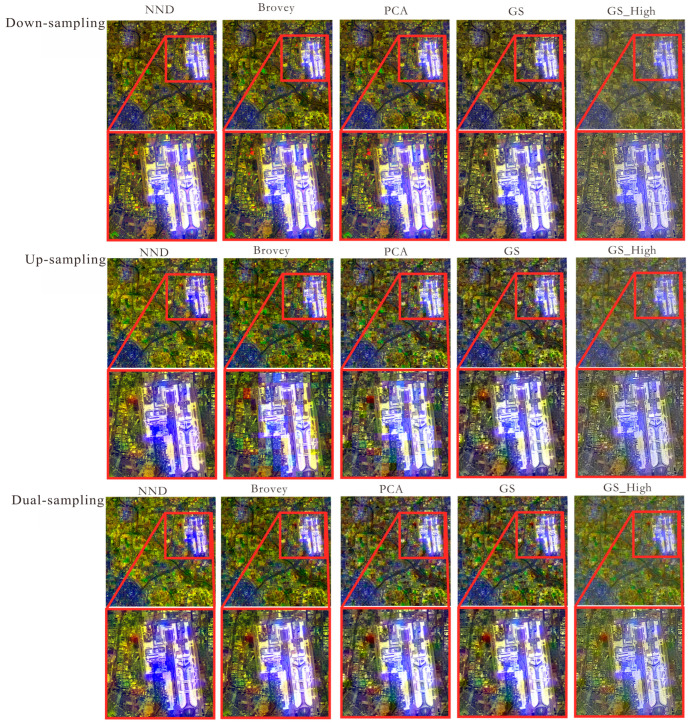
Comparison of color nighttime light remote sensing images with different sampling methods.

Based on the down-sampling multispectral image, the 500 m color nighttime light remote sensing image is up-sampled to the 30 m level matching the original multispectral image and then fused with the 15 m panchromatic image to generate the color nighttime light remote sensing image. The results of the processing are shown in Figure 7.

### 3.3. Image Quality Evaluation

(1) Subjective Evaluation

**Figure 8:**
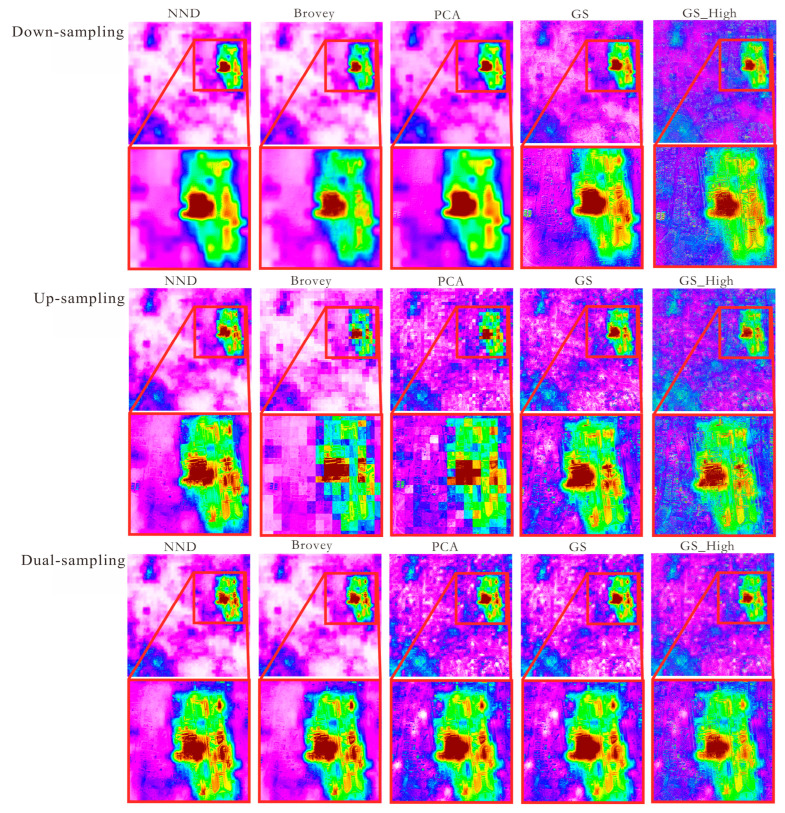
Comparison of third-band images with different sampling methods.

Through visual judgment of Figure 7, it is found that the down-sampling method retains better spectral information than the other methods, but it makes the loss of regional edge light information more serious in the band fusion, and the feature detail information is not as clear as the images obtained by the other two methods. Up-sampling directly fuses the color nighttime light remote sensing image from 500 m to 15 m by using a panchromatic image during image fusion, which is not consistent with the 30 m multispectral image corresponding to the 15 m panchromatic image in the original Landsat 8. The dual-sampling adjustment method can better improve the effect of multi-resolution image fusion while retaining the edge lighting information of the 500 m nighttime light remote sensing image. In order to further verify the advantages of the dual-sampling adjustment method, its third band is compared, as shown in Figure 8.

The red square area clearly shows that the spatial detail of NND, Brovey, PCA, GS, and GS_High has been enhanced by the dual-sampling adjustment. This is especially true for the edges of buildings, residential areas, and river water bodies, where the contours are clearer. Compared with GS and GS_High, the images transformed using NND, Brovey, and PCA are blurred in low-light areas, which makes it impossible to visualize the detailed textures of the features. In terms of spectral retention effect, the texture of GS_High features is more obvious than that of GS.

(2) Objective Evaluation

Standard Deviation (STD), Information Entropy (EN), Average Gradient (AG) correlation coefficient (CC), Spectral Distortion (SD), Peak Signal-to-Noise Ratio (PSNR), and Structural Similarity (SSIM) are used as the main evaluation indicators. This is shown in Table 1 below.

**Figure 9:**
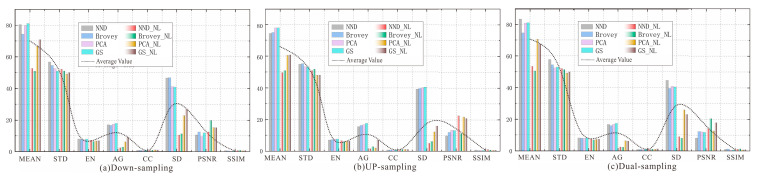
Comparison of quality evaluation of ‘up–down dual sampling’ fusion image.

The images generated by the three sampling methods were evaluated using the above indexes, with the results shown in Figure 9.

**Figure 10:**
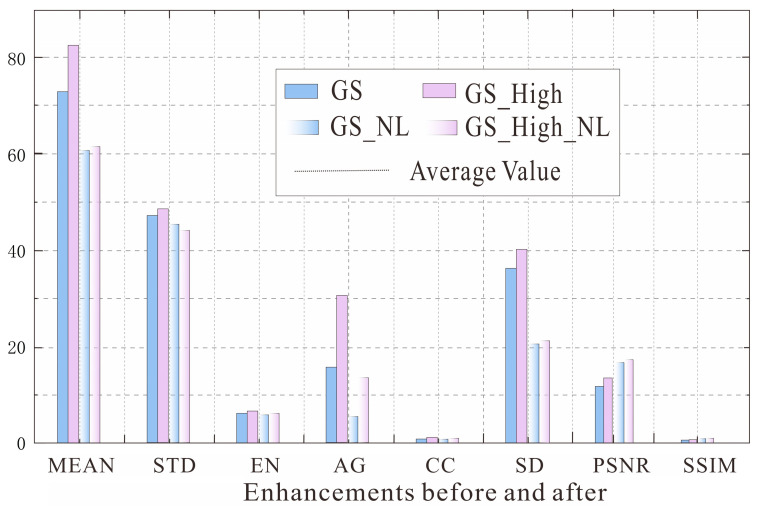
Quality evaluation before and after image enhancement.

The down-sampled image has the highest AG, with GS performing the best and Brovey performing the best in terms of STD. In addition, CC and SSIM are the lowest and have the largest distortion. The up-sampled image has the highest CC and PSNR. It is lower in terms of MEAN, STD, EN, and AG, but the GS and PCA transforms perform better in these aspects. The images generated by using dual-sampling adjustment and their third band perform best in terms of MEAN, STD, EN, CC, and PSNR; especially, the evaluation indicators of NDD, Brovey, PCA, and GS transforms are all improved. This indicates that the image adjusted by using dual-sampling performs better in terms of the average brightness and pixel dispersion and has the best ability to maintain spectral features. Among them, the indicators processed by GS are more stable, so a high-pass filter algorithm is used for enhancement, as shown in Figure 10.

Compared with GS transformation, the color nighttime light remote sensing imagery obtained by GS_High has obvious improvement with each index of the third band, so GS_High is selected for image fusion to generate color nighttime light remote sensing imagery and is used for the analysis of urban functional types.

## 4. Dual-Sampling Adjustment to Generate Color Nighttime Light Remote Sensing Imagery

### 4.1. Research Data Analysis

**Figure 11:**
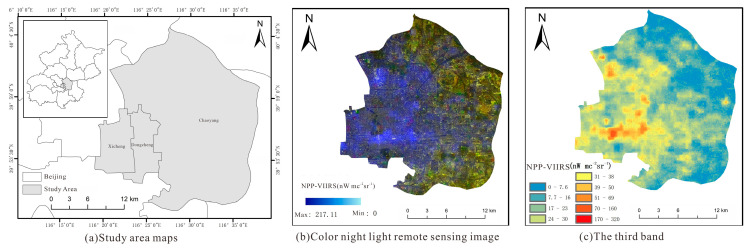
Study area data types.

Chaoyang District, Dongcheng District, and Xicheng District of Beijing were selected as the research areas. These three areas are located in the central urban districts of Beijing, covering different socio-economic characteristics, including commercial centers, historical and cultural heritage areas, and high-end residential areas. This provides a deeper understanding of the living conditions of urban residents and the level of economic development, as shown in Figure 11a–c.

**Figure 12:**
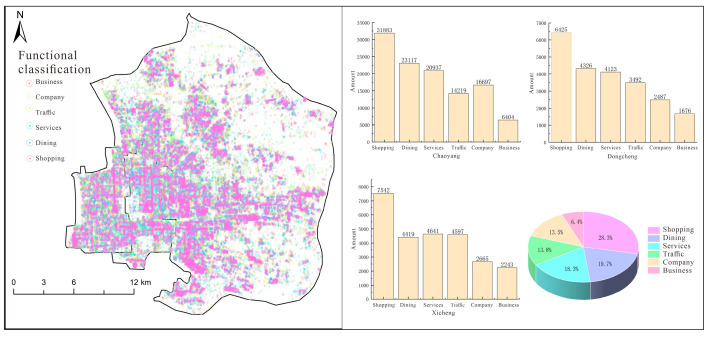
Distribution of urban functional types and statistics.

In order to show the distribution of urban functional types more clearly, this paper shows the distribution of urban functions in three counties and districts by using points. It is difficult to distinguish different urban functional types using only different colors. Therefore, the fourteen urban functional types were sorted, and the top six functional types were extracted to obtain the distribution results of urban functional types in the city center. The urban function situation of the three city centers was also counted, as shown in Figure 12.

Shopping is the main functional type in the city center, occupying a quarter of the city’s area, and it is mainly distributed in the southwestern region. Additionally, Chaoyang District, located in the northeastern part of Beijing, has a large number of shopping malls, office buildings, and corporate enterprises, so the traffic facilities are relatively lower. Dongcheng District and Xicheng District are located in the central urban area of Beijing, adjacent to Tiananmen Square and the Forbidden City. They are the political and cultural centers of Beijing, so traffic facilities are prioritized over companies and businesses.

### 4.2. Kernel Density Analysis of POIs in Urban Functional Areas

Kernel density analysis is an assessment index that represents the probability of spatial geographic events occurring within a certain area [28]. It can intuitively reflect the distribution of discrete values within a continuous area and can be used in research fields such as analyzing urban functional types and urban spatial structure division [29]. The formula is as follows:

(2) $$p_{i}(x)=\frac{1}{n}\sum_{i=1}^{n}k_{h}(x-x_{i})$$

**Figure 13:**
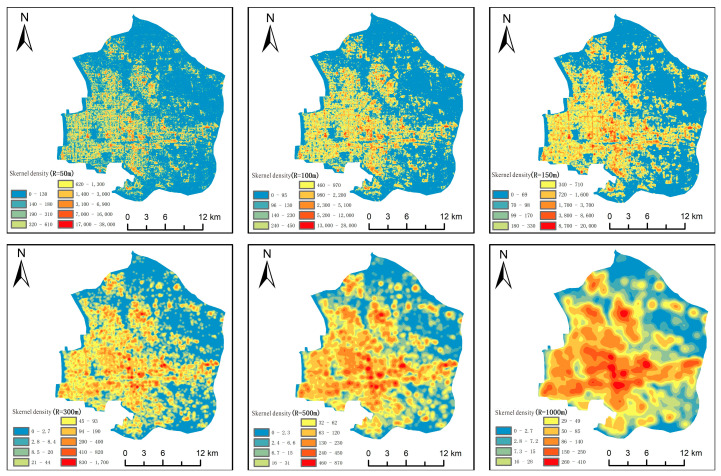
Kernel density analysis results.

In the formula, $p_{i}(x)$ is the kernel density function and $k_{h}(x)=\frac{1}{n}k(\frac{x}{h})$ is the scaling function of the kernel density function. h is the smoothing parameter of the bandwidth, and n is the total number of samples. An appropriate bandwidth R has a significant impact on the research results. Therefore, a bandwidth of 50–1000 m was chosen, as shown in Figure 13.

**Figure 14:**
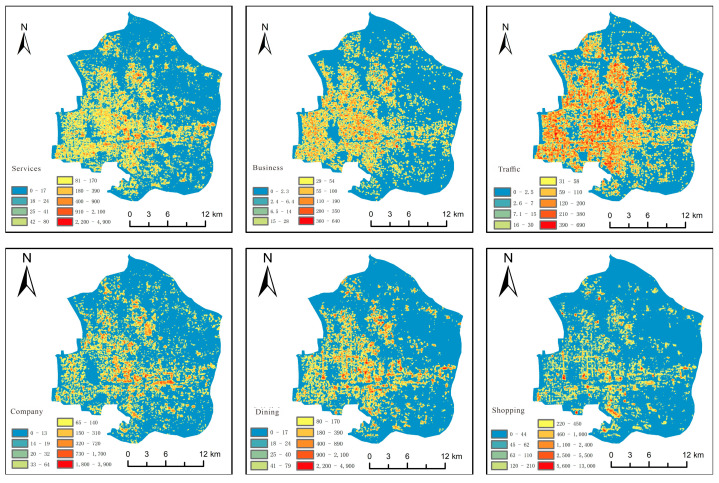
Distribution of urban functional types.

By comparing Figure 13, it was found that the density center is more stable between bandwidths of 100–150 m. Therefore, 150 m was chosen as the bandwidth for kernel density, with a pixel size of 15 m. Then, the six urban functional types were analyzed, and the results are shown in Figure 14.

### 4.3. Urban Functional Correlation Analysis Based on Color Light Values

Before conducting the Pearson correlation analysis, the image was log-transformed. The method from reference [30] was used to process the fused imagery and POI kernel density analysis results. Then, Pearson correlation analysis was performed separately on the nighttime light values and kernel density results of the three regions. The formula is as follows:

(3) $$r=\frac{\sum_{i=1}^{m}\left(x_{i}-\bar{x}\right)\left(y_{i}-\bar{y}\right)}{\sqrt{\sum_{i=1}^{m}{\left(x_{i}-\bar{x}\right)}^{2}\sum_{i=1}^{m}{\left({xy}_{i}-\bar{y}\right)}^{2}}}$$

where r is the Pearson correlation coefficient; the value range is [−1, 1]; m is the total number of samples; $x_{i}$ and $y_{i}$ represent the observed values of the two variables; $\bar{x}$ and $\bar{y}$ represents the mean values of the two variables.

**Figure 15:**
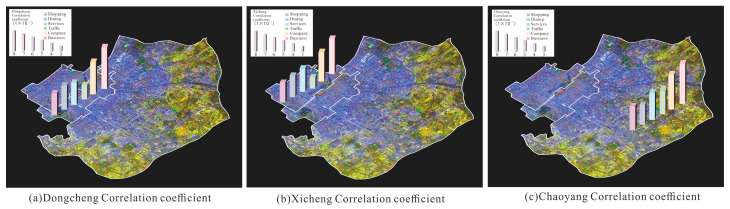
Correlation analysis between color nighttime light values and POIs.

Current studies predominantly focus on correlating nighttime light values with overall urban POI data, yet limited attention has been given to urban functional types at finer spatial scales. To address this gap, we conducted a correlation analysis between color nighttime light values across three distinct zones and six urban functional types. This approach aims to expand the applicability of high-resolution color nighttime light remote sensing imagery in small-scale urban planning research. The results are shown in Figure 15.

As shown in Figure 15, the nighttime light brightness in the three study areas exhibits positive correlations with kernel density analysis results, indicating that color nighttime light remote sensing imagery effectively reflect urban night economic activity. Specifically, color nighttime light values show the highest correlation coefficients with businesses (r ≥ 0.7221), followed by companies (r = 0.6120). This phenomenon can be attributed to the economic centrality of these regions, where frequent night business activities drive strong correlations. In Chaoyang District, a high-end commercial zone in Beijing, the relatively dispersed distribution of dining areas results in a weaker correlation with color nighttime light values. Conversely, traffic in Dongcheng and Xicheng Districts demonstrates lower correlations with light brightness (r = 0.4217 and 0.3027, respectively), primarily due to their status as historical and cultural heritage zones, which restricts infrastructure development and reduces interdependencies.

In summary, the dual-sampling adjustment method not only enhances image quality and analytical precision but also strengthens the practicality and validity of this study. By enabling a more accurate identification of urban functional types, urban planners can allocate resources rationally and formulate targeted policies to improve residents’ quality of life and promote sustainable urban development.

## 5. Conclusions

This study addresses the resolution disparity between daytime and nighttime light remote sensing imagery by proposing a dual-sampling adjustment method to enhance color nighttime light imagery. The effectiveness of the method in preserving image details and improving spectral quality is validated through comprehensive subjective and objective evaluations. Key findings are summarized as follows:

(1) The dual-sampling adjustment method generates color nighttime light imagery with a spatial resolution improved from 500 m to 15 m and spectral bands expanded from single-band to three bands (red, green, blue). This approach retains nighttime light distribution while incorporating daytime surface features, significantly improving the comprehensive performance of nighttime light remote sensing imagery.
(2) Subjective and objective evaluations demonstrate that the dual-sampling adjustment image, particularly its third band, achieves the best performance in indicators such as MEAN, STD, EN, CC, and PSNR. These results confirm the method’s superiority in preserving spatial textures, enhancing information capacity, and maintaining spectral fidelity.
(3) Urban functional type analysis reveals that the enhanced color nighttime light remote sensing imagery accurately captures urban spatial features. The brightness of color nighttime light exhibits the strongest correlation with businesses, followed by companies, providing novel data support for dynamic monitoring of urban functions.

## 6. Discussion

This study addresses the challenges of low-resolution nighttime light remote sensing imagery and the significant resolution discrepancies in daytime and nighttime image fusion by proposing an innovative dual-sampling adjustment method to enhance color nighttime light remote sensing imagery. This provides a novel perspective for multi-source remote sensing fusion and urban planning applications. However, this study has limitations. First, the study area (Dongcheng, Xicheng, and Chaoyang Districts in Beijing) may constrain the generalizability of the findings. Second, the lack of a standard for image quality assessment introduces some variability in different evaluation indicators; additionally, seasonal variations in light intensity and the dynamic nature of urban economies may influence the analysis of urban functional types. Future work will expand the study range, adopt comprehensive evaluation indicators, and investigate urban functional types from spatiotemporal perspectives. Furthermore, the dual-sampling adjustment method will be optimized to enhance robustness in complex environments, and its application potential will be explored in urban planning, ecological surveys, and environmental monitoring.

## Figures and Tables

**Table 1:** Evaluation of indicator performance introduction.

Evaluation Metric	Performance
MEAN	Reflects the overall brightness level of the image, characterizing the distribution of sensitivity.
STD	Represents color contrast by quantifying pixel value dispersion; higher values indicate stronger contrast.
EN	Measures the richness and integrity of spectral information; higher entropy denotes greater informational diversity.
AG	Evaluates detail clarity, with higher gradients corresponding to sharper edges and textures.
CC	Assesses spectral consistency between the image and a reference; values approaching 1 indicate superior fidelity.
SD	Quantifies spectral distortion severity; lower values signify enhanced fidelity.
PSNR	Integrates spectral fidelity and detail preservation; higher PSNR reflects improved image quality.
SSIM	Holistically evaluates brightness, contrast, and texture in comparison to a reference image; values closer to 1 denote higher alignment.

## Data Availability

Data are contained within the article.
